# Thermostable Artificial Enzyme Isolated by *In Vitro* Selection

**DOI:** 10.1371/journal.pone.0112028

**Published:** 2014-11-13

**Authors:** Aleardo Morelli, John Haugner, Burckhard Seelig

**Affiliations:** Department of Biochemistry, Molecular Biology, and Biophysics, University of Minnesota, Minneapolis, Minnesota, United States of America, & BioTechnology Institute, University of Minnesota, St. Paul, Minnesota, United States of America; University of Toulouse - Laboratoire d'Ingénierie des Systèmes Biologiques et des Procédés, France

## Abstract

Artificial enzymes hold the potential to catalyze valuable reactions not observed in nature. One approach to build artificial enzymes introduces mutations into an existing protein scaffold to enable a new catalytic activity. This process commonly results in a simultaneous reduction of protein stability as an undesired side effect. While protein stability can be increased through techniques like directed evolution, care needs to be taken that added stability, conversely, does not sacrifice the desired activity of the enzyme. Ideally, enzymatic activity and protein stability are engineered simultaneously to ensure that stable enzymes with the desired catalytic properties are isolated. Here, we present the use of the *in vitro* selection technique mRNA display to isolate enzymes with improved stability and activity in a single step. Starting with a library of artificial RNA ligase enzymes that were previously isolated at ambient temperature and were therefore mostly mesophilic, we selected for thermostable active enzyme variants by performing the selection step at 65°C. The most efficient enzyme, ligase 10C, was not only active at 65°C, but was also an order of magnitude more active at room temperature compared to related enzymes previously isolated at ambient temperature. Concurrently, the melting temperature of ligase 10C increased by 35 degrees compared to these related enzymes. While low stability and solubility of the previously selected enzymes prevented a structural characterization, the improved properties of the heat-stable ligase 10C finally allowed us to solve the three-dimensional structure by NMR. This artificial enzyme adopted an entirely novel fold that has not been seen in nature, which was published elsewhere. These results highlight the versatility of the *in vitro* selection technique mRNA display as a powerful method for the isolation of thermostable novel enzymes.

## Introduction

Protein stability is often a limiting factor for the application, engineering and structural studies of proteins. Low protein stability can result in aggregation, susceptibility to protease degradation and poor yields in the expression of soluble protein, thereby complicating the study and use of these proteins. For commercial applications, proteins commonly need to be particularly stable to increase their tolerance to process conditions like high temperatures or organic solvents [1]. Furthermore, proteins with low stability are less tolerant to mutations thereby limiting further engineering because even slightly destabilizing mutations can lead to unfolding. This can create situations where mutations that would improve enzyme activity in a protein engineering project appear ineffective because the enzyme was not stable enough to remain folded [2]. Conversely, improved thermal stability correlates with mutational robustness and evolvability [3].

Methods to increase the thermodynamic stability of proteins include rational design, consensus-based design, directed evolution, and commonly some combination of these approaches [4]. Rational design introduces mutations predicted to enable additional stabilizing interactions [5]. However, this approach requires extensive structural knowledge, substantial computing power and is technically challenging, which still limits the accessibility of this method. Consensus based-design utilizes phylogenetic information to determine which amino acids are preferred at certain positions [5]. This method can be used to reconstruct thermostable ancestral proteins or, be combined with structural knowledge, which likely further improves the prediction of stabilizing mutations. However, these approaches are dependent on the quality of the constructed phylogenetic tree, which is non-trivial to accurately assemble. Directed evolution is a combinatorial approach that introduces mutations at random and then screens for desired properties such as improved activity or stability [6]–[8]. High throughput screens are often performed *in vivo*, utilizing colorimetric [9] or fluorescent [10] reporters to measure levels of soluble expression as readout for stability, or *in vitro* using protease resistance and phage display [11], [12]. Protein variants are also commonly assayed directly for thermostability and activity as purified proteins, but these methods have a relatively low throughput. Numerous examples have recently been discussed in excellent review articles [4], [13]. As mutations are introduced randomly, the chance of success increases with the number of mutants sampled. This favors high throughput methods which can sample millions to trillions of mutants [14], [15]. Individual methods aimed to generate more stable protein variants can also be combined for best results as was demonstrated by consensus design that used the sequence output of a library selection [16].

We previously reported the *in vitro* selection of *de novo* RNA ligase enzymes that catalyze a reaction not observed in nature [17]. These artificial enzymes ligate RNA with a 5′-triphosphate to the 3′-hydroxyl of second RNA forming a native 5′-3′ linkage and releasing pyrophosphate. These artificial ligases are zinc dependent metalloenzymes of about 10 kDa. Several enzymes resulting from this *in vitro* selection experiment were analyzed in more detail. All examined enzymes were soluble when expressed as fusion proteins with maltose-binding protein (MBP), but most enzymes were poorly soluble when expressed on their own. NMR HSQC spectroscopy of the most soluble clone, ligase #6, revealed that a significant portion of the protein was well-folded, yet the overall resolution of the data was insufficient to solve the three-dimensional structure [17]. To overcome this issue, we again utilized *in vitro* selection. We modified the conditions of our original procedure and continued the selection to isolate ligase variants with improved stability in order to facilitate structural and mechanistic studies of these artificial enzymes.

Here, we describe in detail the *in vitro* selection of RNA ligases with increased stability. For this directed evolution experiment we utilized the mRNA display technology, an *in vitro* display method, which covalently links each protein to its encoding mRNA [18], [19]. Using this technology, up to 10^13^ unique proteins can be sampled in a single experiment, which is orders of magnitude more than most other selection strategies [14]. To isolate enzymes with increased thermodynamic stability, we modified parts of the selection procedure and performed the ligation step at 65°C. For the selection reported here, we used the output library from our previous selection at room temperature [17] as starting material without further diversification. We hypothesized that enzymes, which are active at elevated temperature, will have a more stable protein fold that in turn will facilitate structural characterization. We also hoped that the increased structural stability would correspond to increased solubility and expression *in vivo*. After several rounds of selection, representative ligase clones were sequenced and tested for soluble expression in *E. coli.* The soluble and most active ligase 10C was characterized further and its activity and stability was compared to two closely related sequences from the previous selection at room temperature. The experiments revealed that ligase 10C is both more stable and more active than either of these ligases. We recently described the three-dimensional structure of ligase 10C solved by NMR, revealing a novel fold that has not been observed in nature and lacks secondary structural elements like α-helices or β-strands [20]. Furthermore, we reported a detailed analysis of the substrate specificity of ligase 10C showing that this enzyme can facilitate the selective isolation and sequencing of any RNA with a 5′-triphosphate [21].

This manuscript is the first report of an mRNA display selection at high temperature. These results demonstrate the efficacy of mRNA display for isolating thermostable enzymes as stability and activity are selected simultaneously in a high throughput experiment.

## Materials and Methods

### Preparation of Oligonucleotides

^32^P-labeled PPP-substrate-23 used in the original selection at 23°C (5′-PPP–GGAGACUCUUU) and PPP-substrate-65 for the selection at 65°C (5′-PPP–GGAGAUUCACUAGCUGGUUU) were prepared through T7 transcription as reported previously [17], [22]. The HO-substrate-23 (5′–CUAACGUUCGC), HO-substrate-65 (5′-UCACACUGUCUAACGUUCGC) and HO-substrate-65-Bio (5′-(PC)-UCACACUGUCUAACGUUCGC, (PC) represents PC biotin phosphoramidite from Glen Research, Sterling, VA) were purchased from Dharmacon (Lafayette, CO) and prepared according to the manufacturer's protocol. The DNA splint (5′–GAGTCTCCGCGAACGT) complementary to the substrates-23, and RNA splint (5′- AAACCAGCUAGUGAAUCUCCGCGAACGUUAGACAGUGUGA) complementary to the substrates-65, were purchased from Integrated DNA Technologies (Coralville, IA). The reverse transcription primer (HEG_4_-RT) was produced by ligating the PPP-substrate-65 to BS75P-HEG_4_ in the presence of BS76 as template using T4 DNA ligase [23] and purified by denaturing PAGE. All oligonucleotides were dissolved in ultra-pure water and concentrations determined by UV absorbance.

### Selection of RNA Ligases at 65°C

The mRNA display selection was performed as previously published [17], with the following exceptions. Primers BS99 and BS24RXR2 were used to amplify the DNA by PCR. Primer BS99 replaces the N-terminal FLAG affinity tag that was used in the previous selection at room temperature [17] with the E-tag. Accordingly, both FLAG affinity purification steps in the previous protocol were substituted by E-tag affinity purifications. For the first E-tag purification, the mRNA-displayed proteins eluted from the oligo(dT)cellulose were mixed with binding buffer (same as Flag binding buffer [17]) and then incubated for 30 min at 4°C with rotation with 25 µL Anti-E affinity gel (from Anti E-tag affinity column, GE healthcare Biosciences; prewashed with E clean buffer (100 mM glycine, pH 3.0, 0.05% Tween-20) and binding buffer). The Anti-E tag affinity gel was then washed with binding buffer and eluted with binding buffer containing two equivalents of E-peptide (Bachem, Osteocalcin (7–19, human); one equivalent of E-peptide saturates the antigen sites of the antibody resin) for 3 min at 4°C. The second E-tag purification was performed in a similar fashion using 50 µL Anti-E affinity gel and 6 equivalents of E-peptide to elute. The elution from the second E-tag affinity purification was incubated with the HO-substrate-65-Bio and the RNA splint in presence of 2 mM MgCl_2_ and 100 µM ZnCl_2_ for 1 hour at 65°C in selection rounds 1, 2, 3 and 5. In round 4, the sample was divided into two aliquots, one of which was incubated for 1 h, and the other aliquot was incubated for 5 min. The reaction was quenched and purified on streptavidin beads as described previously [17], and the photocleaved DNA was amplified by PCR and used as input for the following round. For the starting material in round 5, the photocleaved DNA from round 4 was used that resulted from the 5 min incubation.

### Expression & Purification of RNA Ligases

RNA ligases were expressed and purified as previously described [20].

### Screening for Ligase Activity by Gel-Shift Assay

5 µM ^32^P-labeled PPP-substrate-65, 6 µM RNA splint, 7 µM HO-substrate-65, 20 mM HEPES pH 7.5, 100 mM NaCl, 100 µM ZnCl_2_ and 1.7 µM enzyme (purified by Ni-NTA affinity chromatography [20]) were combined and incubated for 16 hours at 23°C and 65°C. Reactions were stopped by the addition of EDTA to a final concentration of 10 mM. Immediately following, the RNA was denatured for 40 min at 65°C in 7.5% formaldehyde, 58% formamide and 11.6 mM MOPS pH 7.0. Samples were separated by 20% denaturing PAGE gel containing 2% formaldehyde. The gel was analyzed using the GE Healthcare (Amersham Bioscience) Phosphorimager and ImageQuant software (Amersham Bioscience). The amount of radiation in both the substrate and product bands was measured and the percentage of ligation was determined by dividing the intensity of the product band by the sum of the product and substrate bands.

### Determination of Observed Rate Constants (k_obs_)

5 µM enzyme (purified by Ni-NTA affinity and size exclusion chromatography [20]) was incubated with 10 µM ^32^P-labeled PPP-substrate-23, 15 µM DNA splint, 20 µM HO-substrate-23 and ligation was monitored for up to 2 hours at 23°C. Reactions were quenched with two volumes of 20 mM EDTA in 8 M urea after 0, 15, 30, 60 and 120 minutes, heated to 95°C for 4 min and separated by 20% denaturing PAGE gel. The gel was analyzed using the GE Healthcare (Amersham Bioscience) Phosphorimager and ImageQuant software (Amersham Bioscience). The rate constant (k_obs_) was calculated by determining the slope of the linear fit of percentage of ligation over time and correcting for enzyme concentration by multiplying with the ratio of PPP-substrate to enzyme (2 = 10 uM/5 uM) resulting in a value with the unit h^−1^. The reported values are an average of 3 independent replicates ± the standard deviation. Total conversion was <10% for all cases.

### Circular Dichroism and Thermal Denaturation

Ligase enzymes (purified by Ni-NTA affinity and size exclusion chromatography [20]) were concentrated to 50 µM and dialyzed against CD buffer (150 mM NaCl, 2 mM HEPES, 0.5 mM 2-mercaptoethanol, 100 µM ZnCl_2_). Circular dichroism spectra and thermal denaturation curves were recorded on a JASCO J-815 spectropolarimeter at 30 µM or 50 µM protein, respectively. The following parameters were used for both measurements: 1.5 nm band width, 2 seconds response time, standard sensitivity, 10 accumulations. The ellipticity at 222 nm was monitored to determine thermal denaturation curves over a temperature range from 5 to 91°C with a ramp rate of 1°C/min and a temperature pitch of 2°C.

## Results

### Setup of Selection Procedure

**Figure 1:**
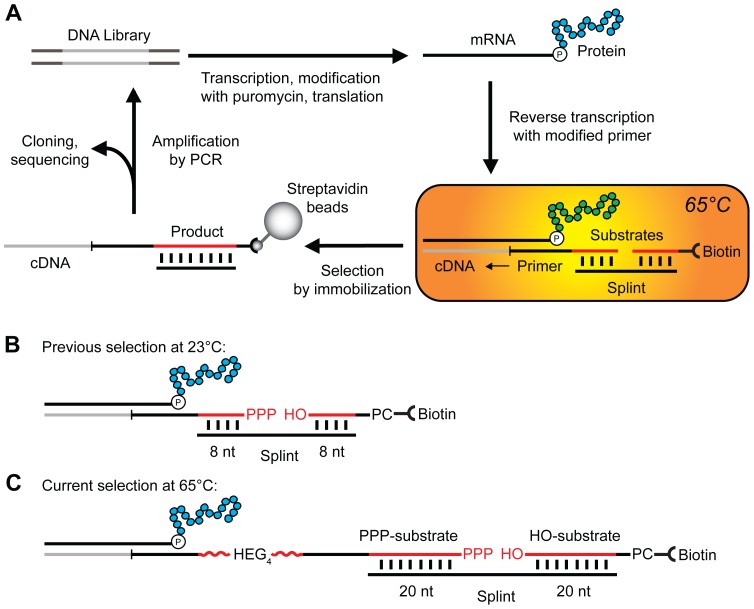
*In vitro* selection of artificial ligase enzymes with increased stability. (**A**) Schematic of the isolation of ligase enzymes. The DNA library encodes the library of proteins that resulted from the original selection of ligase enzymes at 23°C [17], [22]. The DNA is transcribed into RNA, modified with puromycin at the 3′-end and translated *in vitro* yielding a library of mRNA-displayed proteins [22]. Reverse transcription with a primer containing one RNA substrate shown in red results in a complex of protein, mRNA, cDNA and substrate. This complex is incubated at 65°C with the second RNA substrate (red) and the complementary splint as highlighted in the orange box. The cDNA of ligases active at this temperature is immobilized on streptavidin beads and amplified for subsequent rounds of selection, or identified by cloning and sequencing. (**B**) Detailed view of ligation reaction substrates in complex with the mRNA-displayed protein. The two strands of RNA in red, the 5′-triphosphate RNA (PPP-substrate) and 3′-hydroxyl RNA (HO-substrate), are joined in a template-dependent ligation reaction. The PPP-substrate is part of the reverse transcription primer. The photocleavable site (PC) is used to release the cDNA that encodes active enzymes from streptavidin immobilization by irradiation at 365 nm. The splint acts as template of the ligation and base pairs with 8 nucleotides of each RNA substrate during the previously published selection at 23°C [17], [22], and with (**C**) 20 nucleotides of each substrate during the current selection at 65°C. HEG_4_ represents the linker of four hexaethylene glycol units (red wavy line).

Sequence analysis of the artificial RNA ligase enzymes that resulted from the final round of the previous *in vitro* selection performed at 23°C [17] revealed substantial sequence diversity. The DNA encoding those diverse ligases was used as the starting library for the selection at 65°C described in this paper without introducing further sequence diversity. The RNA ligation reaction catalyzed by the previously selected enzymes was dependent on a complementary splint oligonucleotide that base-pairs to the two substrate RNAs [17] (Figure 1). During the selection at 23°C, this splint base-paired to eight nucleotides of each substrate (Figure 1B). In order to ensure stable base-pairing during a splinted ligation at elevated temperatures, a longer splint was chosen to extend the region complementary to each substrate to twenty nucleotides (Figure 1C). The 40-nucleotide-long splint resulted in a melting temperature of 76°C and 69°C with the PPP-substrate and the HO-substrate, respectively (Figure S1).

To enable the selection of active enzymes, the PPP-substrate was linked to the mRNA-displayed proteins via the reverse transcription (RT) primer that initiates the cDNA synthesis (Figure 1A). This linkage resulted in a high local concentration of substrate in the vicinity of each protein. In order to reduce this local concentration and thereby favor the selection of enzymes with an increased substrate affinity, we lengthened the RT primer by an additional eighteen non-complementary nucleotides and four flexible hexaethylene glycol linker units (HEG_4_, Figure 1C). The hexaethylene glycol linker simply acted as a long unstructured tether to increase the average distance between protein and substrate. The use of the longer RT primer in combination with the splint of 40 nucleotides (nt) in length (Figure 1C) resulted in a ligase activity of about 50% compared to a ligation using the shorter RT primer and the 16 nt splint (Figure 1B).

We then evaluated the ligase activity of the starting library at increasing temperatures in order to determine a temperature at which the majority of the library members are inactive. Using the 40 nt splint and the HEG_4_-RT primer, at 65°C no ligation was detectable (<10%), whereas at 60°C the ligation activity was about half of the activity measured at 23°C. Therefore, we decided to carry out the selection for higher stability at 65°C.

During the previous selection for ligases, 57% of the isolated enzymes had acquired a second FLAG binding sequence (DYKXXD) in addition to the FLAG binding sequence that was part of the N-terminal constant region. This was likely a result of a selection bias caused by two FLAG affinity purification steps per round of selection. In order to counteract this FLAG purification bias, we changed the selection protocol to using the E-tag affinity purification instead. Therefore, we replaced the FLAG tag coding sequence in the N-terminal constant region of the library with an E-tag sequence by PCR. The ligation activity was unaffected by the change of tags.

### *In Vitro* Selection at 65°C

**Figure 2:**
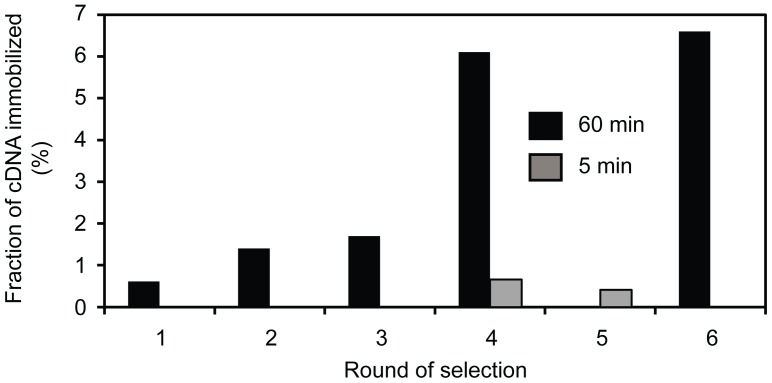
Progress of selection for ligases at 65°C. The fraction of ^32^P-labelled cDNA that bound to streptavidin agarose after each round of selection is shown. The reaction time was either 60 min or 5 min as indicated by black or gray bars, respectively.

To enrich for RNA ligase enzyme with increased thermostability, we performed a total of six rounds of selection and amplification (Figure 1A). After reverse transcription, the mRNA-displayed proteins were incubated with the HO-substrate-65 and the RNA splint for 60 min and/or 5 min. The percentage of cDNA that was immobilized on streptavidin beads after each round of selection is shown in Figure 2. In the case of the 60 minute incubation, the percentage of immobilized cDNA increased steadily over the course of the selection, from 0.61% after round 1 to 6.6% after round 6. In order to increase the selection pressure by favoring enzymes with faster ligation rates, in round 4, we incubated a second aliquot of the mRNA-displayed proteins for only 5 min yielding 0.66% immobilized cDNA. This cDNA was used as input for following round, but no increase in the amount of immobilized cDNA after 5 min incubation was observed in round 5 (amount decreased to 0.41%). Therefore, we performed the sixth and final round of selection, again with 60 min incubation. The resulting DNA was cloned and sequenced for further analysis.

### Sequence Analysis and Expression of Selected Ligases

The sequence alignment of 32 clones from the sixth round of selection at 65°C revealed two protein families (Figure S2). One representative clone from each family was cloned and expressed in *E. coli* to examine soluble expression (Figure S3). While both clones expressed well, ligase 10C was highly soluble whereas ligase 10H was largely insoluble. Furthermore, native Ni-NTA affinity purification of ligase 10H yielded no soluble protein (data not shown) and, therefore, ligase 10H was not characterized further.

**Figure 3:**
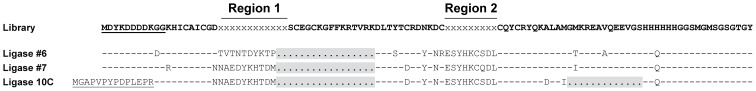
Sequence alignment of the library used as input for the original ligase selection with ligases #6, #7 [17] and 10C that were selected at 23°C and at 65°C, respectively [41], [42]. The amino acids in regions 1 and 2 of the original library (on top) were randomized prior to the selection at 23°C and are shown as “x” [43]. Dashes symbolize amino acids that are identical to the starting library. A period highlighted in gray represents a deletion. The underlined N-terminal amino acids of the library and ligase 10C represent a Flag epitope tag and an E epitope tag, respectively.

The sequence of ligase 10C shared similarities to ligases #6 and #7 from the original selection with #7 being more similar (Figure 3). All three ligases were almost identical in sequence in the formerly randomized region 2, and all three shared the deletion of 17 amino acids following region 1. Ligases 10C and #7 also shared the sequence in region 1, but 10C contained a second deletion of 13 amino acids near the C-terminus. This C-terminal deletion was also found in other clones from the selection at 23°C [17], but these proteins were poorly soluble when expressed without an maltose-binding protein fusion and therefore unsuited for a direct comparison.

### Activity of Ligase Enzymes

**Figure 4:**
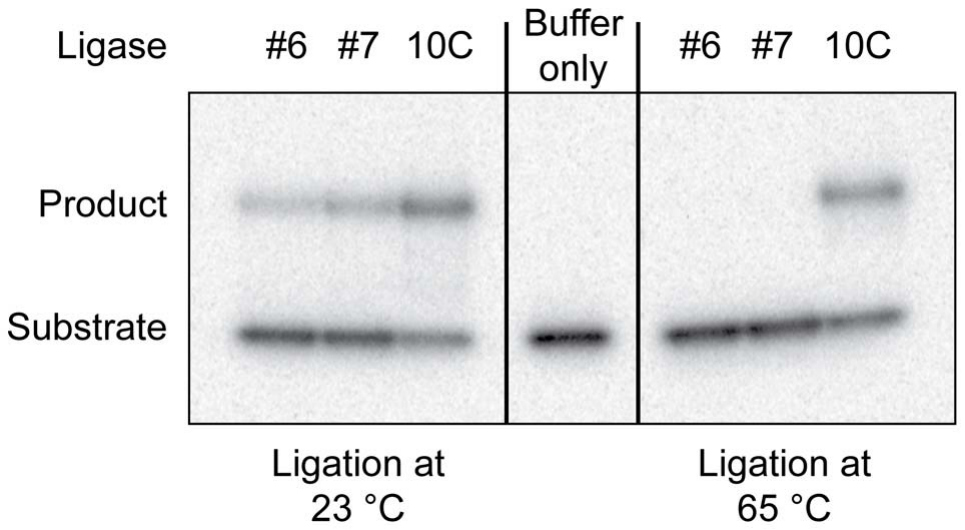
Activity of ligase enzymes assayed at different temperatures. Ligases #6 and #7 had been selected previously at 23°C [17], [22] and ligase 10C was selected at 65°C. In this assay, the ^32^P-labeled PPP-substrate-65, HO-substrate-65 and 40 nt splint were incubated with the individual enzymes for 16 h and the activity was monitored by a gel-shift assay.

To compare the enzymatic activity of ligase 10C to ligases #6 and #7, we assayed the three enzymes at 23°C and 65°C (Figure 4). Ligase 10C was the only enzyme active at 65°C. In comparison, ligases #6 and #7 were active at room temperature as expected, but had no measurable activity at 65°C. In addition to its activity at 65°C, ligase 10C was also active at room temperature. To compare the activity of the three enzymes more accurately, we measured the k_obs_ for each ligase at 23°C. At a subsaturating substrate concentration of 10 µM, ligase 10C had a k_obs_ of 0.165±0.015 h^−1^ while ligases #6 and #7 had k_obs_ of 0.0174±0.0066 h^−1^ and k_obs_ of 0.0207±0.0045 h^−1^, respectively (Table S1). This represents an 8 to 10-fold increased activity of ligase 10C compared to ligases #6 and #7 even at 23°C. While the main goal of the selection was to isolate an enzyme with greater thermostability, as an added benefit, the most stable enzyme also featured an improved catalytic rate at room temperature.

### Characterization of Thermal Stability by Circular Dichroism (CD)

**Figure 5:**
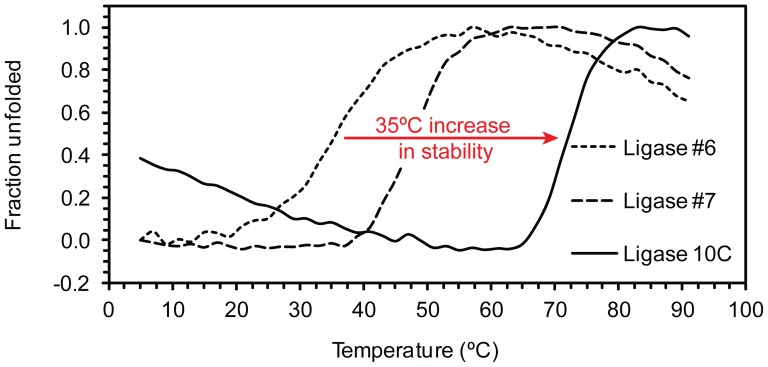
Thermal unfolding curves of ligases #6, #7 and 10C. Thermal unfolding was monitored by circular dichroism at 222 nm. For each measurement 10 accumulations were acquired.

In order to assess if the unique enzymatic activity of ligase 10C at 65°C was correlated to increased structural stability, we measured thermal denaturation curves of all three ligases by circular dichroism. In preparation of the thermal unfolding experiment, we measured the CD spectra of the three enzymes (Figure S4). All three spectra exhibited two minima of negative ellipticity at 205 nm and between 220 and 225 nm, respectively. While those minima suggested α-helical secondary structural content [24], the 205 nm minimum was substantially more negative than the second minimum, which differs from purely alpha helical proteins that have similar absolute values for both minima. In fact, the three-dimensional structure of ligase 10C recently solved by NMR revealed that α-helices and β-strand regions are essentially absent in ligase 10C [20]. Nevertheless, we used the strong negative ellipticity of all three ligases at 222 nm to monitor thermal unfolding of the proteins over a temperature range from 5 to 91°C. We found all three enzymes to give the characteristic single sigmoidal transition corresponding to a two-state unfolding reaction (Figure 5). As determined from the curves, the enzymes showed very different melting temperatures. Ligase 10C had the highest melting temperature (T_m_ = 72°C), which was 35 degrees higher than the T_m_ of ligase #6 (37°C), and 24 degrees higher than the T_m_ of ligase #7 (48°C). The high melting temperature of 72°C for ligase 10C was in agreement with its retained enzymatic activity at 65°C as the enzyme had not undergone unfolding yet. In contrast, ligases #6 and #7 were fully denatured at 65°C, and, therefore, their complete lack of enzymatic activity at 65°C could be explained by their unfolding.

## Discussion

We isolated a thermostable artificial RNA ligase enzyme by *in vitro* selection at 65°C of a library of artificial ligases that were originally generated at 23°C. The isolated ligase 10C was more thermostable and more active than the two most closely sequence-related ligases #6 and #7 identified during the selection at 23°C. Ligase 10C had a melting temperature (T_M_) of 72°C corresponding to a stability increase of 24 degrees compared to #7, and 35 degrees compared to ligase #6. Previously reported T_M_ improvements through protein engineering are commonly between 2 to 15 degrees [5]. The T_M_ increase by 35 degrees reported here favorably compares with those rare examples of ‘record-setting stabilizations’ [4], [25]–[27]. While the ligases #6 and #7 had no measurable enzymatic activity at 65°C, ligase 10C ligated RNA at 65°C with an activity that was similar to its activity at 23°C. Furthermore, the activity of ligase 10C at 23°C was about an order of magnitude higher than the activity of the ligases #6 and #7 at the same temperature.

**Figure 6:**
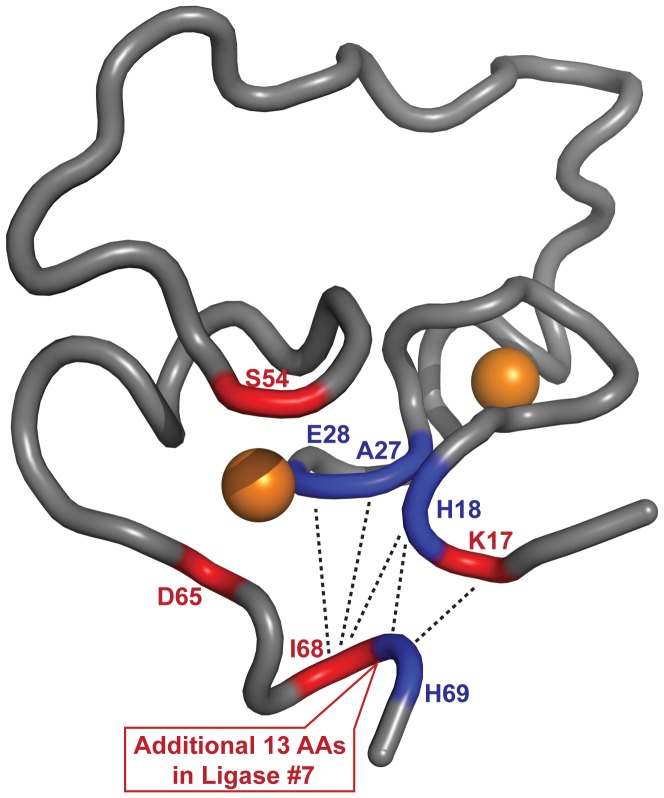
Sequence differences between ligase #7 and ligase 10C mapped onto the NMR structure of ligase 10C [20]. Mutations are shown in red. Residues potentially perturbed by the mutations are labeled in blue and long range NOEs are shown as dashed black lines. The two coordinated zinc ions as depicted as orange spheres and the residue numbers refer to ligase 10C. The unstructured termini of ligase 10C were omitted for clarity.

The increased thermostability of ligase 10C was likely due to additional intramolecular contacts within the protein compared to the mesophilic ligases #6 and #7. In contrast to these enzymes isolated at 23°C, the properties of ligase variant 10C were suitable to solve its three-dimensional solution structure by NMR [20]. The structure featured a small, well-folded core coordinated by two Zn^2+^-ions. In addition, the folding core also contained a highly dynamic internal loop and was framed by unstructured termini. In order to discuss a potential correlation between differences in primary sequence and altered thermal stability, we mapped sequence differences between ligase #7 and 10C onto the structure of 10C (Figure 6). We chose ligase #7 for comparison because despite the high sequence similarity it showed a large difference in thermostability. All sequence differences between these two ligases were found in or near the structured region responsible for zinc coordination. We previously demonstrated by NMR that residues Ile68 and His69 near the C-terminus of ligase 10C made long range NOE contacts with several residues at the N-terminus (Lys17, His18, Ala27 and Glu28) [20]. Notably, His18 was one of the zinc coordinating residues in ligase 10C [20] and mutating this position to Ala resulted in a drastically reduced solubility of the protein. In ligase #7, the residue corresponding to Ile68 was a methionine. In addition, ligase #7 contained an additional 13 amino acids located between the residues corresponding to Ile68 and His69 in ligase 10C, which likely moved His69 and prevented its contacts with Lys17, His18 at the N-terminus. Presumably, all these mutations could compromise the intramolecular interactions in these positions reported for ligase 10C and decrease the stability of ligase #7 at high temperature. Ligase 10C also differed from ligase #7 in two additional positions (Ser54 and Asp65) which may further influence protein stability. A direct comparison of the overall flexibility of ligase 10C and the two mesophilic ligases would require solving also the structures of ligases #6 and #7 by NMR. This would be beyond the scope of this paper and preliminary experiments suggested that ligase #6 is not amenable to detailed NMR studies.

The *in vitro* selection at 65°C not only yielded the family A of related sequences that included ligase 10C (Figure S2), but also a second family B represented by ligase 10H which could not be expressed solubly in *E. coli*. During the original selection at 23°C, we noted that of the seven ligases characterized, only #6 and #7 were soluble without being expressed as a MBP fusion. While ligase 10C was closely related to ligases #6 and #7, ligase 10H is most similar to ligase #1, which also did not express solubly. Isolating proteins like ligase 10H and ligase #1 is not surprising because mRNA display uses a eukaryotic *in vitro* translation system and therefore soluble expression in *E. coli* was never directly selected for. Additionally, the covalently linked RNA increases protein solubility which can also contribute to this result. In general, this solubilizing effect is a favorable feature of mRNA display because it allows identifying proteins that might be lost during other selection techniques due to poor solubility. It is possible that ligase 10H could be solubilized through MBP fusion like ligase #1, but such a modification would have complicated subsequent structural studies.

Considering the high melting temperature of 72°C for ligase 10C, it is particularly surprising to discover the lack of secondary structural motifs like α-helices or β-strands combined with highly dynamic regions [20]. The structure of this artificial enzyme does appear to match with any known protein folds. While it is increasingly appreciated that catalytic activity of enzymes can require conformational flexibility [28]–[30], thermal stability is usually associated with tight packing and rigidity. Generally, thermophilic enzymes possess well packed hydrophobic cores [31], few exposed surface loops [32] and additional stabilizing interactions such as salt bridges [33] and a high number of hydrogen bonds [34]. These features lead to an increased rigidity that, while favoring stability at higher temperature, often appears to decrease activity at lower temperature. This observation has been interpreted to mean that stability, dynamics and catalysis are a tradeoff, but this common notion has recently been called into question [35]. The structure of the ligase 10C [20] combines a high flexibility and the absence of a packed hydrophobic core with thermostability, and is equally active at 65°C and at ambient temperature. The structure of this *de novo* enzyme challenges the common view of how enzymes are supposed to look – a view that is biased by proteins amenable to crystallization.

The high degree of disorder and flexibility present in ligase 10C might be a feature that favors its evolvability. For example, the presence of disordered regions and loosely packed structures found in viral proteins, structural characteristics similar to those found in ligase 10C, may allow for increased evolvability because each mutation, due to a lower amino acid interconnectivity, would lead to a slower loss in stability, compared to the more packed structures of thermophilic enzymes [36]. Similarly, ligase 10C might also be highly evolvable because of its flexible structure and disordered regions. Yet, this artificial enzyme was generated *de novo* and, unlike biological proteins, has not been shaped by billions of years of evolution. As its structure and function has just come into existence, ligase 10C could be considered a model protein for primordial enzymes. For these reasons, properties of this enzyme like its evolutionary potential will be interesting to study, however comparisons to natural proteins might be challenging.

The starting library for this selection at elevated temperature was a mixture of protein variants that was the final output of the previously described selection for artificial ligases at 23°C [17]. No further genetic diversity had been introduced. Sequencing of the starting library showed a diverse mixture of unrelated sequences and sequence families. Ligase 10C had not been observed during the sequencing of 49 individual clones and was only sufficiently enriched and detected after the subsequent selection at 65°C. It is conceivable that future mutagenesis and directed evolution of ligase 10C using the same selection strategy will further improve thermal stability and activity. These studies will help us understand the evolutionary potential of this artificial enzyme and also yield improved catalysts for a variety of applications [21].

The discovery of this thermostable enzyme and its unusual structure emphasizes the value of directed evolution approaches that do not require a detailed understanding of protein structure-function relationships, but instead randomly sample sequence space for functional proteins. In contrast, it would have been impossible to construct this particular artificial enzyme by rational design despite recent advances in rational protein engineering. In the current project, we employed the *in vitro* selection technique mRNA display [18], [19]. This method uses product formation as the sole selection criterion and is independent of the mechanism of the catalyzed reaction. The technique has several advantages over other selection strategies [37]. The mRNA display technology enables to search through large libraries of up to 10^13^ protein variants. This feature is beneficial because the chance of finding a desired activity increases with the number of variants interrogated. Furthermore, the *in vitro* format of this method allows selecting for activity under a wide range of conditions, which is similar to the common approach of screening much smaller libraries of purified proteins, but in contrast to *in vivo* selection strategies where maintenance of cell viability limits the experimental possibilities. Previous reports on mRNA display include the improvement of folding and stability of proteins by selecting for resistance to protease degradation [38], or by selecting in the presence of increasing amounts of the denaturant guanidine hydrochloride [39], [40]. Interestingly, in parallel to our successful selection for RNA ligases at elevated temperature, we also attempted a similar selection in presence of guanidine hydrochloride, but no enrichment was observed even after six rounds (data not shown). Nevertheless, to our knowledge the work presented here is the first description of an mRNA display selection at elevated temperatures yielding thermostable proteins. The *in vitro* format of mRNA display should facilitate other selections at a variety of pH, temperatures, ionic strength, or in the presence of co-solvents, inhibitors or other chemicals. Such experiments will help to study the coevolution of protein stability and activity, and also has the potential to produce proteins that are more stable in industrial or biomedical applications.

## Supporting Information

Figure S1 **Thermal denaturation of substrate and splint oligonucleotides used in the selection and activity assays at 65°C.** The first derivative of the melting curve for the 40 nt splint in the presence of both PPP-substrate-65 and HO-substrate-65 RNA oligonucleotides is presented. The concentration of each oligonucleotide was 0.5 µM in a buffer containing 70 mM KCl, 100 µM ZnCl_2_, 5 mM 2-mercaptoethanol and 20 mM HEPES at pH 7.5.
(TIF)

Figure S2 **Clones identified from round 6 of the** ***in vitro*** **selection at 65°C.** Two protein families (A, B) were identified and a representative clone from each family was chosen for further characterization (ligase 10C and ligase 10H, shown in red).
(TIF)

Figure S3 **Protein expression in** ***E. coli*** **of representative ligases selected at 65°C.** A Coomassie-stained SDS-PAGE gel shows samples of whole cells pre- and post-induction and the insoluble and soluble fractions after cell lysis and centrifugation. Red boxes in the lane ‘Soluble fraction’ indicate the presence or absence of soluble ligases 10C and 10H, respectively.
(TIF)

Figure S4 **Circular dichroism spectra of ligases #6, #7 and 10C at 25°C.**
(TIF)

Table S1 Data for determining k_obs_.
(DOCX)

Table S2 Sequences of ligases 10C and 10H selected at 65°C; and ligases #6 and #7 selected previously at 23°C for comparison.
(DOCX)

Table S3 Sequences of oligonucleotides.
(DOCX)
