# Supplementary figures and images for: Thermostable Artificial Enzyme Isolated by In Vitro Selection
Source: PLoS One. 2014 Nov 13;9(11):e112028. doi: 10.1371/journal.pone.0112028 (PMC4230948; doi:10.1371/journal.pone.0112028)

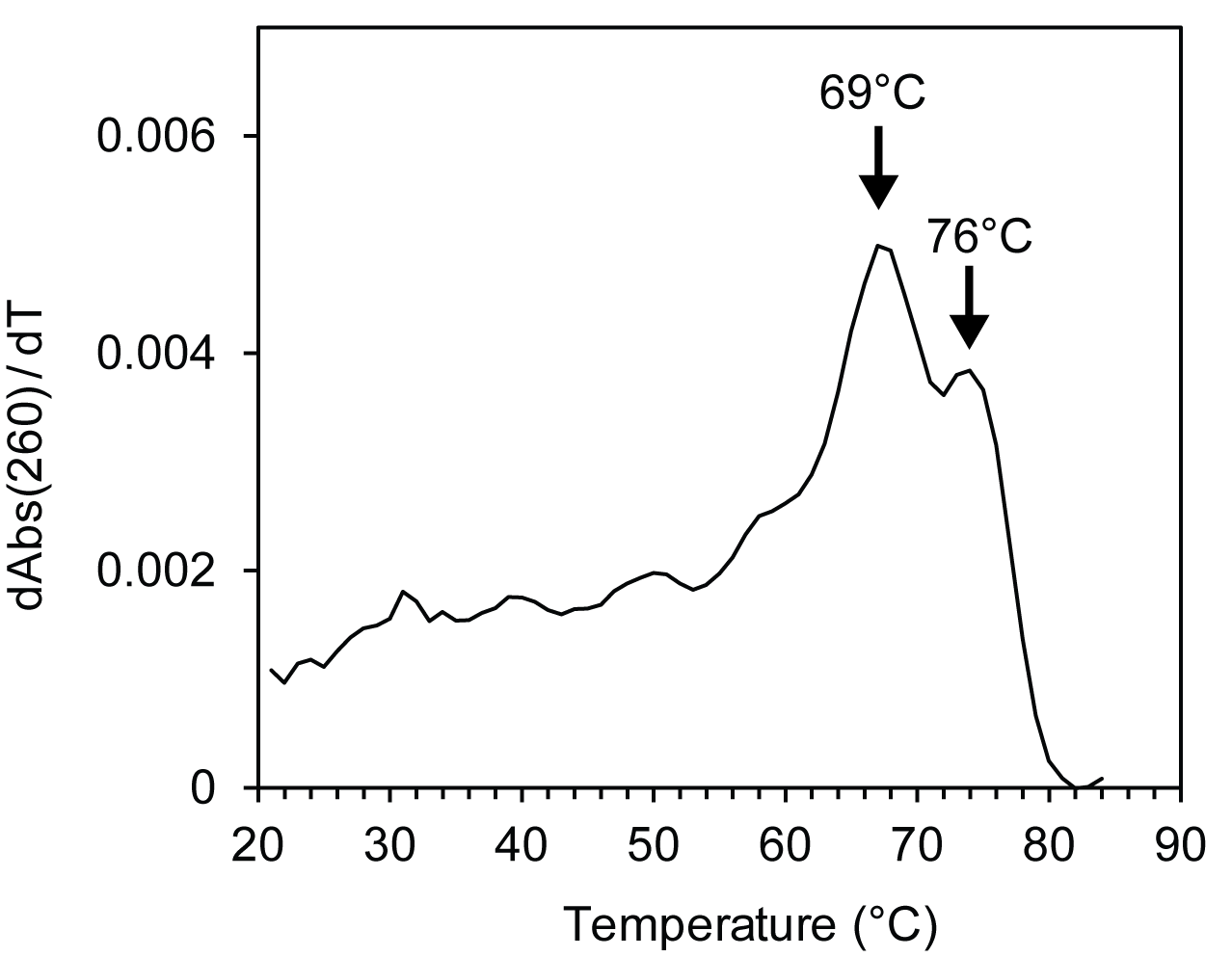

Supplement: Figure S1 — Thermal denaturation of substrate and splint oligonucleotides used in the selection and activity assays at 65°C. The first derivative of the melting curve for the 40 nt splint in the presence of both PPP-substrate-65 and HO-substrate-65 RNA oligonucleotides is presented. The concentration of each oligonucleotide was 0.5 µM in a buffer containing 70 mM KCl, 100 µM ZnCl2, 5 mM 2-mercaptoethanol and 20 mM HEPES at pH 7.5. (TIF) [file pone.0112028.s001.tif]

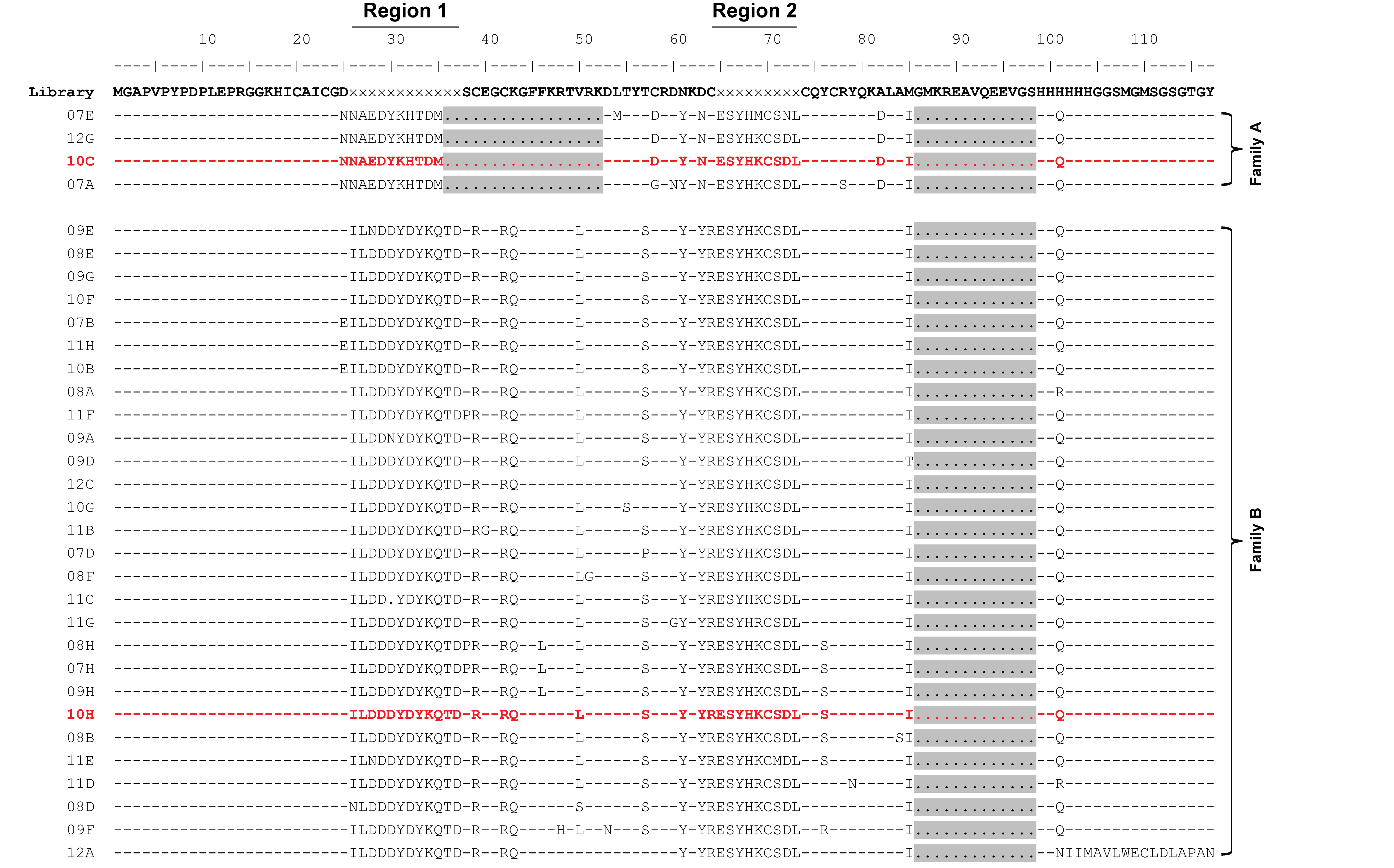

Supplement: Figure S2 — Clones identified from round 6 of the in vitro selection at 65°C. Two protein families (A, B) were identified and a representative clone from each family was chosen for further characterization (ligase 10C and ligase 10H, shown in red). (TIF) [file pone.0112028.s002.tif]

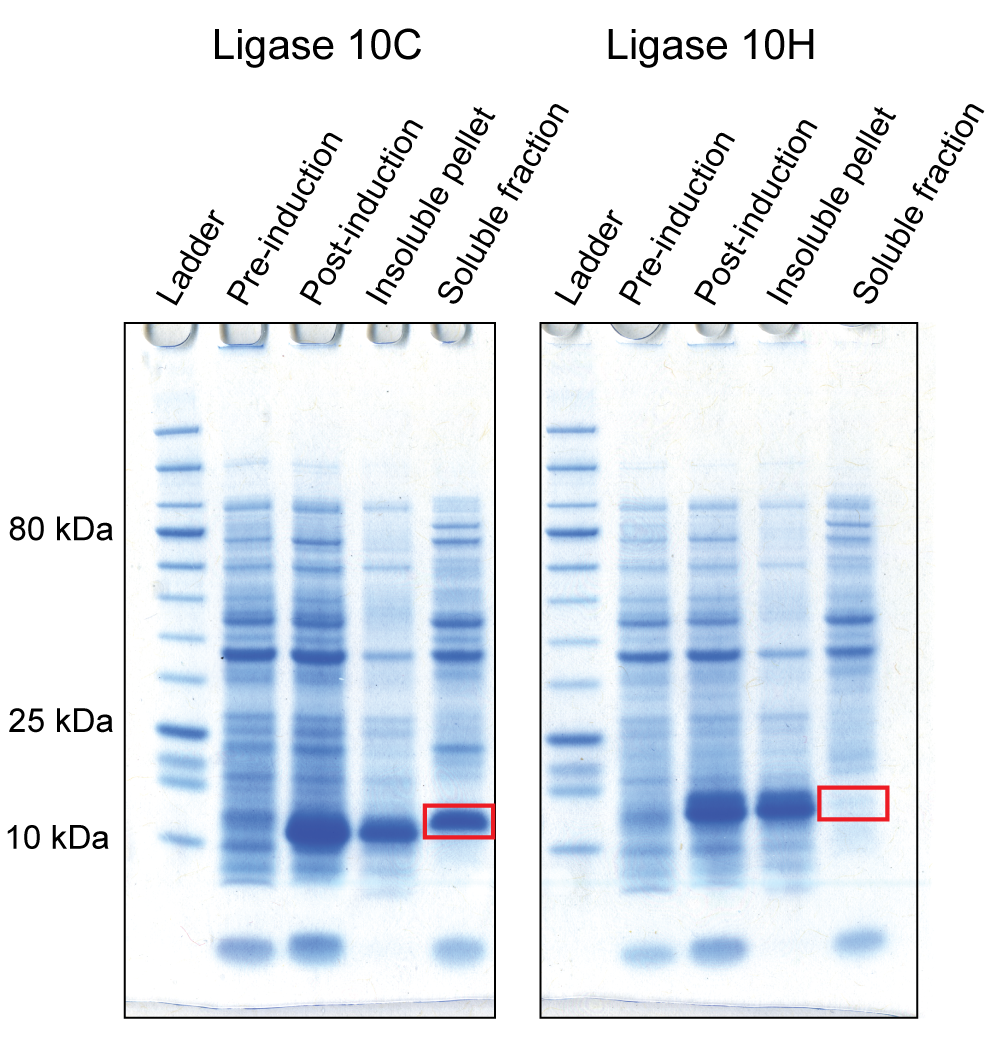

Supplement: Figure S3 — Protein expression in E. coli of representative ligases selected at 65°C. A Coomassie-stained SDS-PAGE gel shows samples of whole cells pre- and post-induction and the insoluble and soluble fractions after cell lysis and centrifugation. Red boxes in the lane ‘Soluble fraction’ indicate the presence or absence of soluble ligases 10C and 10H, respectively. (TIF) [file pone.0112028.s003.tif]
