# Supplementary material for: Thermostable Artificial Enzyme Isolated by In Vitro Selection
Source: PLoS One. 2014 Nov 13;9(11):e112028. doi: 10.1371/journal.pone.0112028 (PMC4230948; doi:10.1371/journal.pone.0112028)
Supplement: Table S1 — Data for determining kobs. (DOCX) [file pone.0112028.s005.docx]

| **Replicate** | **Ligase 10C** | | **Ligase #6** | | **Ligase #7** | |
| --- | --- | --- | --- | --- | --- | --- |
|  | **Slope*** | **R^2^** | **Slope** | **R^2^** | **Slope** | **R^2^** |
| 1 | 0.0894 h^-1^ | 0.973 | 0.0124 h^-1^ | 0.910 | 0.0128 h^-1^ | 0.989 |
| 2 | 0.0828 h^-1^ | 0.982 | 0.00768 h^-1^ | 0.951 | 0.00984 h^-1^ | 0.993 |
| 3 | 0.0750 h^-1^ | 0.981 | 0.00599 h^-1^ | 0.834 | 0.00840 h^-1^ | 0.991 |

**Table S1. Data for determining k_obs_**. * Five timepoints were used for ligases #6 and #7. Only the first four timepoints were used for ligase 10C as the final point had begun to plateau and would have skewed the analysis.
