# Supplementary material for: Thermostable Artificial Enzyme Isolated by In Vitro Selection
Source: PLoS One. 2014 Nov 13;9(11):e112028. doi: 10.1371/journal.pone.0112028 (PMC4230948; doi:10.1371/journal.pone.0112028)
Supplement: Table S2 — Sequences of ligases 10C and 10H selected at 65°C; and ligases #6 and #7 selected previously at 23°C for comparison. (DOCX) [file pone.0112028.s006.docx]

| **Ligase 10C** | MGAPVPYPDPLEPRGGKHICAICGNNAEDYKHTDMDLTYTDRDYKNCESYHKCSDLCQYCRYQKDLAIHHQHHHGGSMGMSGSGTGY |
| --- | --- |
| **Ligase 10H** | MGAPVPYPDPLEPRGGKHICAICGDILDDDYDYKQTDSREGRQGFFKRTLRKDLTYSCRDYKYRESYHKCSDLCQSCRYQKALAIHHQHHHGGSMGMSGSGTGY |
| **Ligase #6** | MDYKDDDDKDGKHICAICGDTVTNTDYKTPDLTSTCRDYKNRESYHKCSDLCQYCRYQKALAMGTKREAAQEEVGSHHQHHHGGSMGMSGSGTGY |
| **Ligase #7** | MDYKDDDDKGGRHICAICGNNAEDYKHTDMDLTYTDRDYKNCESYHKCQDLCQYCRYQKALAMGIKREAVQEEVGSHHQHHHGGSMGMSGSGTGY |

**Table S2. Sequences of ligases 10C and 10H selected at 65 °C; and ligases #6 and #7 selected previously at 23 °C for comparison.**
