# Supplementary material for: Thermostable Artificial Enzyme Isolated by In Vitro Selection
Source: PLoS One. 2014 Nov 13;9(11):e112028. doi: 10.1371/journal.pone.0112028 (PMC4230948; doi:10.1371/journal.pone.0112028)
Supplement: Table S3 — Sequences of oligonucleotides. (DOCX) [file pone.0112028.s007.docx]

| **BS75P-HEG_4_** | 5'‑P‑TGTACGATTCGATGACGA‑HEG_4‑_‑TTTTTTTTTTTTTTTCCCAGATCCAGACATTC (“P” represents the 5'-phosphate group, “HEG_4_” represents four hexaethylene glycol units (Spacer18 from Glen Research, Sterling, VA) |
| --- | --- |
| **BS76** | 5'-TCGTCATCGAATCGTACAAAACCAGCTAGTGAATC |
| **BS99** | 5'‑TCTAATACGACTCACTATAGGGACAATTACTATTTACAATTACAATGGGAGCACCAGTCCCTTACCCTGATCCGCTGGAACCGCGTggcggaaagcacatctgc |
| **BS24RXR2** | 5'‑TTAATAGCCGGTGCCAGATCCAGACATTCCCATAGAACCGCCATGATGATG |

**Table S3. Sequences of oligonucleotides.**
